# Efficacy and safety of primary posterior capsulotomy in combined phaco-vitrectomy in rhegmatogenous retinal detachment

**DOI:** 10.1371/journal.pone.0213457

**Published:** 2019-03-08

**Authors:** Kyung-Sup Shin, Hye-Jin Park, Young-Joon Jo, Jung-Yeul Kim

**Affiliations:** Department of Ophthalmology, Chungnam National University Hospital, Chungnam National University College of Medicine, Daejeon, Republic of Korea; University of Warmia, POLAND

## Abstract

**Objective:**

To evaluate the efficacy and safety of posterior capsulotomy by analyzing the long-term visual outcomes in patients with rhegmatogenous retinal detachment (RD), who underwent combined phaco-vitrectomy with or without primary posterior capsulotomy.

**Methods:**

A retrospective longitudinal cohort analysis was performed by using data of rhegmatogenous RD patients undergoing combined phaco-vitrectomy. Patients were divided into two groups; Group A (68 eyes of 68 patients) with capsulotomy, and Group B (39 eyes of 39 patients) without capsulotomy. We reviewed the best-corrected visual acuity (BCVA), incidence of posterior capsule opacification (PCO), clinical features at the diagnosis of rhegmatogenous RD, and intraoperative or postoperative complications following posterior capsulotomy.

**Results:**

The modified BCVA measured by the logarithm of the minimum angle of resolution at initial diagnosis and 3, 6, and 12 months after surgery was 0.67 in Group A versus 0.85 in Group B (p = 0.258), 0.40 in Group A versus 0.50 in Group B (p = 0.309), 0.27 in Group A versus 0.45 in Group B (p = 0.055), and 0.21 in Group A versus 0.47 in Group B (p = 0.014), respectively. In subgroup with macula-on RRD, Group A exhibited better visual outcomes compared to Group B at 6(0.17 versus 0.40 [p = 0.037]) and at 12 months(0.14 versus 0.39 [p = 0.030]). The incidence of PCO in Group B was higher than Group A(28.2% versus 4.4% (p < 0.001)). There were no complications associated with posterior capsulotomy.

**Conclusions:**

A primary posterior capsulotomy during combined phaco-vitrectomy using a 23-gauge vitreous cutter was a safe and effective surgical procedure in patients with RRD patients for preventing postoperative intraocular lens-related PCO.

## Introduction

With the advent of modern instrumentation, improved surgical techniques, and improved intraocular lens (IOL), the incidence of posterior capsule opacification has decreased after cataract surgery, but it still remains the most common cause of visual loss.[1, 2] To maximize the visual recovery of outpatients, posterior capsule opacification can be easily treated with non-surgical neodymium:yttrium aluminum garnet (Nd:YAG) laser capsulotomy. However, this procedure is associated with a small risk of complications such as vitreous floaters, a rise in intraocular pressure, macular edema, and damage and decentration of the IOL.[3–8]

Several approaches for the prevention of posterior capsule opacification, such as a modification of the inflammatory response, a change in intraocular lens design, the proper size of continuous curvilinear capsulorhexis(CCC), and biological targets have been suggested. Nonetheless, no method completely prevents or eliminates the onset of posterior capsule opacification(PCO).[9] Clinicians have also performed posterior continuous curvilinear capsulotomy, which has been frequently used in pediatric cataract surgery for the management of PCO. Although several modified techniques have produced successful results, the outcomes are still dependent on the skill of the clinician, so its wider use is still limited.[10–13]

Since its introduction in the early 1990s, combined phaco-vitrectomy has been frequently used because it can decrease treatment costs and shorten the postoperative recovery time, thus making the procedure more safe and effective. Therefore, the combined procedure has been popularized for the treatment of vitreoretinal disease and combined clinically advanced cataract, particularly in elderly and diabetic patients and during intraocular tamponade.[14–16] Previous studies have reported that several risk factors (e.g., diabetes, rhegmatogenous RD, gas tamponade, and postoperative positioning) affect the development of PCO following combined phaco-vitrectomy.[17–19]

Although previous studies have reported the efficacy and stability of posterior capsulotomy using a vitreous cutter during combined phaco-vitrectomy in patients with a variety of retinal diseases,[20, 21] there have been no reports regarding the clinical outcomes in rhegmatogenous RD patients with a high risk of PCO. Here, we report the long-term visual outcomes of primary posterior capsulotomy together with its efficacy and safety in rhegmatogenous RD patients who underwent combined 23-gauge sutureless phaco-vitrectomy with or without primary posterior capsulotomy.

## Methods

### Subjects

This was a retrospective, observational, comparative study. The study protocol was approved by the institutional review board of Chungnam National University Hospital (Daejeon, Republic of Korea) and adhered to the tenets of the Declaration of Helsinki. The requirement for obtaining informed patient consent was waived due to the retrospective nature of the study.

The patients were evaluated between March 2012 and March 2015 at the retina clinic of Chungnam National University Hospital (Daejeon, Korea) via retrospective medical record review. A total of 107 rhegmatogenous RD patients (107 eyes), who underwent combined phaco-vitrectomy in our clinic and were followed for at least 12 months, were included in our study. The patients were divided into two groups according to whether or not they had undergone primary posterior capsulotomy using a 23-gauge vitreous cutter during combined phaco-vitrectomy. Group A included 68 patients (68 eyes) with posterior capsulotomy during combined phaco-vitrectomy, and Group B included 39 patients (39 eyes) without posterior capsulotomy during combined phaco-vitrectomy. Data were excluded from patients with ocular trauma, uveitis, proliferative vitreoretinopathy, silicone oil injection after vitrectomy, a follow-up period < 1 year, macular hole retinal detachment, complications (posterior capsule rupture, zonular dialysis, radial tear, etc.) during cataract surgery.

### Surgical techniques

The combined 23-gauge sutureless phaco-vitrectomies were performed under retrobulbar anesthesia by a single surgeon (J.Y.K.). The cataract extraction preceded pars plana vitrectomy after insertion of a trocar. A 2.8 mm clear corneal incision was performed with a superior approach, and an anterior chamber was created after filling with a viscoelastic substance (1.4% sodium hyaluronate). A 4.5 mm diameter CCC of the anterior capsule was performed, followed by cortical cleaving hydrodissection. A standard phacoemulsification was performed and the residual cortex was removed by irrigation/aspiration. A vitrectomy was performed for repair of the rhegmatogenous RD after filling the anterior chamber with the viscoelastic substance. Following cataract removal, a spherical, three-piece, acrylic, foldable IOL (Sensar^®^) with a 6.0 mm optical diameter was inserted into the capsular bag immediately before gas tamponade following the fluid/air exchange. The incision was not sutured and no leakage was found after corneal hydration. Just before the end of the surgery, a vitreous cutter was placed at the center of the capsular bag, and a 4 mm diameter posterior capsulotomy was performed in a circular manner by manipulating the vitreous cutter using 5,000 cuts per minute and a vacuum pressure of 400 mmHg.

### Visual acuity

A follow-up of all eyes was done at the initial diagnosis and at 3, 6, and 12 months after surgery to compare the long-term visual outcomes of the two groups. All of the patients were subjected to preoperative the best-corrected visual acuity (BCVA) measurements using a Snellen chart that was converted into the logarithm of the minimum angle of resolution(log MAR). Because a poor visual prognosis was expected for patients with macula-off retinal detachment, subgroup analyses of patients with macula-on detachment were performed to determine the effects of primary post capsulotomy on visual acuity.

During the follow-up period, significant decrease in visual acuity (defined as a reduction of more than 2 lines on the Snellen chart) was resolved by Nd:YAG laser capsulotomy in patients showing clinically advanced PCO. Because the YAG laser immediately improved visual acuity, it was difficult to show the difference of BCVA between the two groups. Therefore we assumed that the visual acuity of patients who had found PCO were maintained without visual recovery during follow up periods. This visual acuity is defined as modified BCVA. For example, if PCO occurred at 3 months after vitrectomy and the visual acuity was recovered from 20/40 (snellen) to 20/25 after the YAG laser, the visual acuity of that patient was assumed to be a 20/40 in 3, 6 and 12 months.

### Statistical analysis

All of the statistical analyses were performed using SPSS statistical software for Windows, version 18.0 (SPSS, Chicago, IL, USA). The BCVA was compared between Groups A and B. The χ^2^ test, Fisher’s exact test, linear by linear association and the independent *t*-test were used to compare multiple factors between groups. The independent *t*-test was used for parametric data and the Mann-Whitney U test was used for nonparametric data. The PCO-free survival was analyzed using Kaplan-Meier survival curves and tested using the log-rank test. In all of the analyses, differences were considered significant at a p value < 0.05.

## Results

### Baseline characteristics

A total of 107 eyes of 107 patients were enrolled: 68 patients with primary posterior capsulotomy during surgery in Group A, and 39 patients without primary posterior capsulotomy during surgery in Group B. There were no significant differences in age, sex, visual acuity at the first visit, type of cataract, range of rhegmatogenous RD, and the kind of gas used for tamponade during surgery at the time of rhegmatogenous RD diagnosis (all, p > 0.05; Table 1).

**Table 1:** Characteristics of patient groups who underwent combined phaco-vitrectomy for rhegmatogenous retinal detachment.

Characteristics	Group A	Group B	*p-*value
Number of patients (eyes)	68	39	
Age (years, mean ± SD)	53.8±19.2	58.1±18.6	0.262*
Sex (male : female)	38 : 30	20 : 19	0.646^†^
BCVA at first visit (LogMAR, mean ± SD)	0.67±0.74	0.85±0.81	0.258*
Refractive error (S.E., diopters, mean ± SD)	-3.11±4.66	-2.65±4.31	0.614*
Axial length (mm, mean ± SD)	25.14±3.43	25.26±2.19	0.844*
Macular on/off	35/33	18/21	0.597^†^
Cataract type (%)			0.056^§^
NS	33 (48.5)	13 (33.3)	
CO	31 (45.6)	20 (51.3)	
PSC	4 (5.9)	6 (15.4)	
Gas type (%)			0.704^‡^
C3F8	64 (94.1)	36 (92.3)	
SF6	4 (5.9)	3 (7.7)	
Extent of RD (%)			0.995^§^
< ¼	13 (19.1)	6 (15.4)	
¼~1/2	28 (41.2)	19 (48.7)	
½~3/4	20 (29.4)	10 (25.6)	
> ¾	7 (10.3)	4 (10.3)	
Location of break (%)			0.867^§^
Superior	42 (61.8)	27 (69.2)	
Inferior	19 (27.9)	6 (15.4)	
Mixed	7 (10.3)	6 (15.4)	

Group A = performed capsulotomy

SD = Standard deviation

S.E = Spherical equivalent

CO = cortical opacity

RD = retinal detachment

^†^Chi-square test

§Linear by linear association

Group B = Not performed capsulotomy

BCVA = Best corrected visual acuity

NS = nuclear sclerosis

PSC = posterior subcapsular cataract

* Independent *t-*test

^‡^Fisher’s exact test

### Posterior capsule opacification

**Fig 1:**
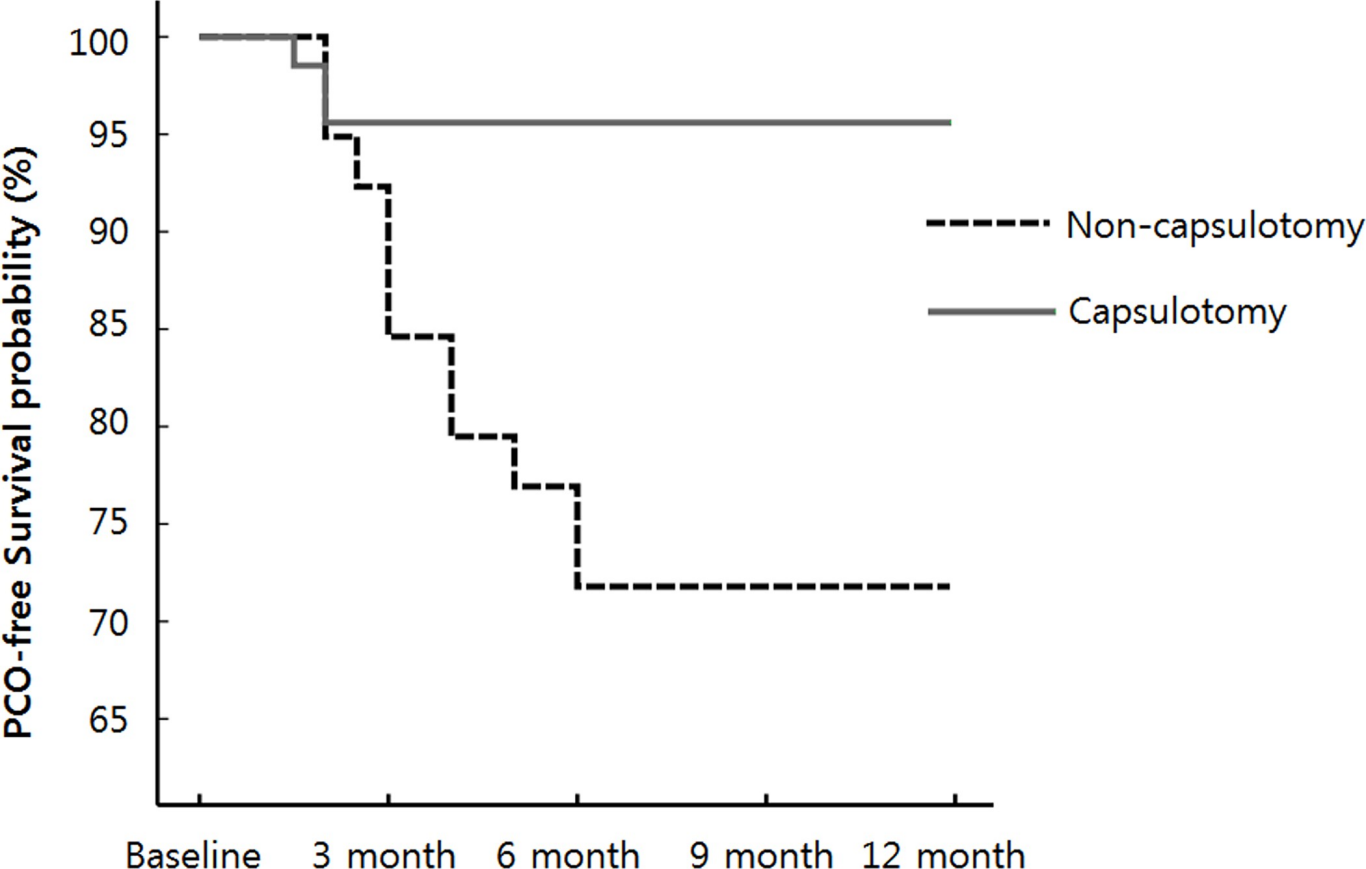
Kaplan-Meier plots showing the posterior capsule opacification (PCO)-free survival probability of the two groups. The PCO percentage was analyzed at 12 months after combined phaco-vitrectomy, involving 4.4% of the patients (3/68 eyes) in Group A treated with primary posterior capsulotomy and 28.2% of the patients (11/39 eyes) in Group B who were not treated with capsulotomy. The percentage difference was statistically significant between the two groups (p < 0.001; log-rank test).

PCO, which caused a significant decrease in visual acuity, occurred in 3 patients (4.4%) in Group A and 11 patients (28.2%) in Group B. There was a greater percentage of PCO in Group B than in Group A (p < 0.01). In Group A, PCO occurred within 2 months (mean, 1.83 ± 0.29 months). The causes of PCO were thin fibrotic membrane (three patients). In Group B, PCO occurred at a mean of 3.68 ± 1.45 months, which was significantly later than occurrence in Group A (p = 0.022). There was no thin fibrotic membrane in Group B. Only typical PCO was observed. All patients with PCO in Group A and B were treated with YAG laser capsulotomy (Table 2; Fig 1).

**Table 2:** The incidence of posterior capsule opacification and intervals in the two groups.

	Group A(n = 68)	Group B(n = 39)	*P*-value
Rates of PCO (%)	3/68(4.4%)	11/39(28.2%)	0.001*
Period to PCO formation, months	1.83±0.29	3.68±1.45	0.022^†^

* Fisher’s exact test

^†^ Mann-Whitney U test

PCO = posterior capsule opacification

**Fig 2:**
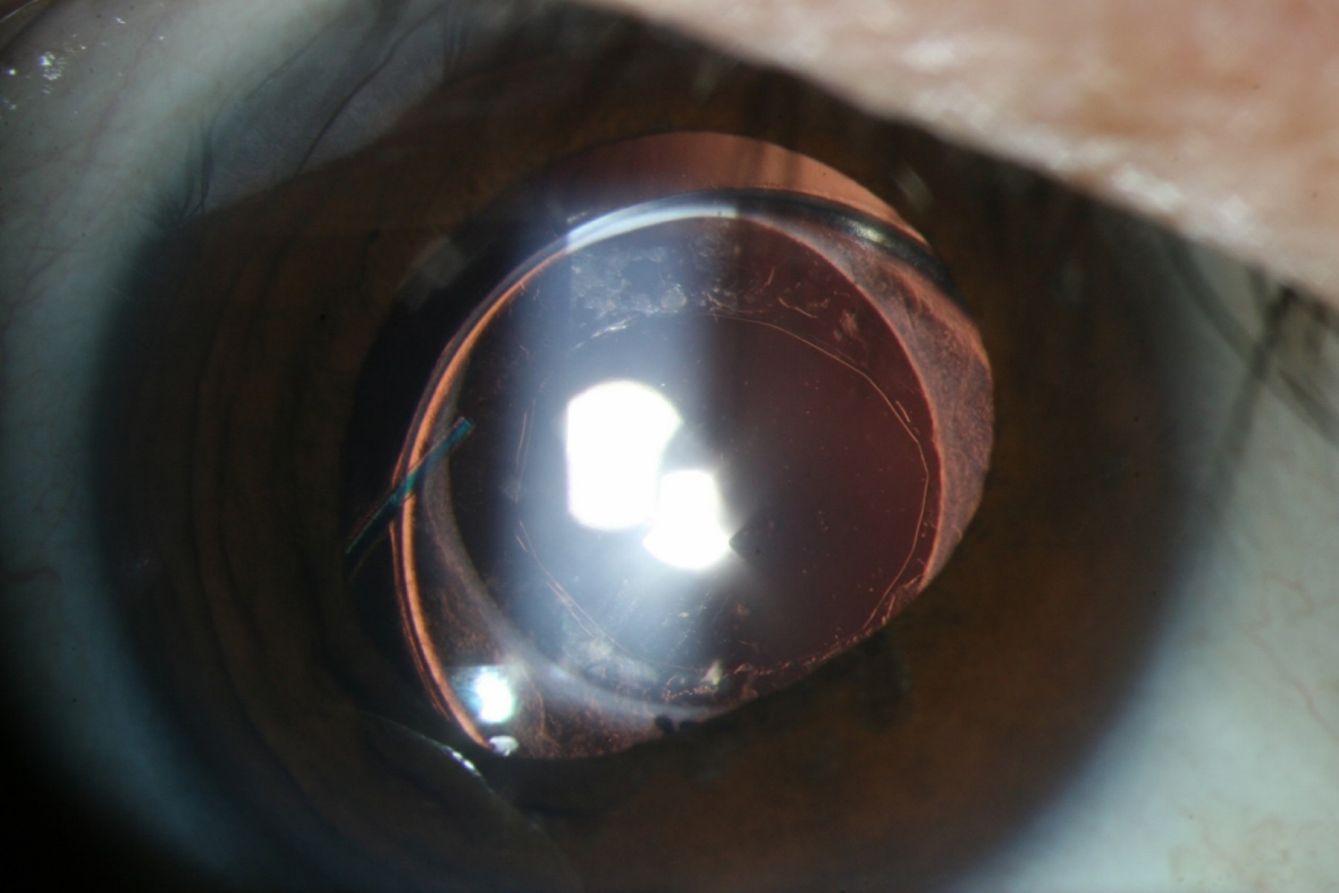
An anterior segment photograph of a 48-year-old male with rhegmatogenous retinal detachment after posterior capsulotomy using 23-gauge combined phaco-vitrectomy. PCO was not detected, with a central optical clear zone over 12 months postoperatively.

There were no cases in which the PCO recurred during the follow-up period. There was a central clear zone until postoperative 1 year in Group A that was treated with primary posterior capsulotomy during the phaco-vitrectomy (Fig 2).

### Visual acuity

**Fig 3:**
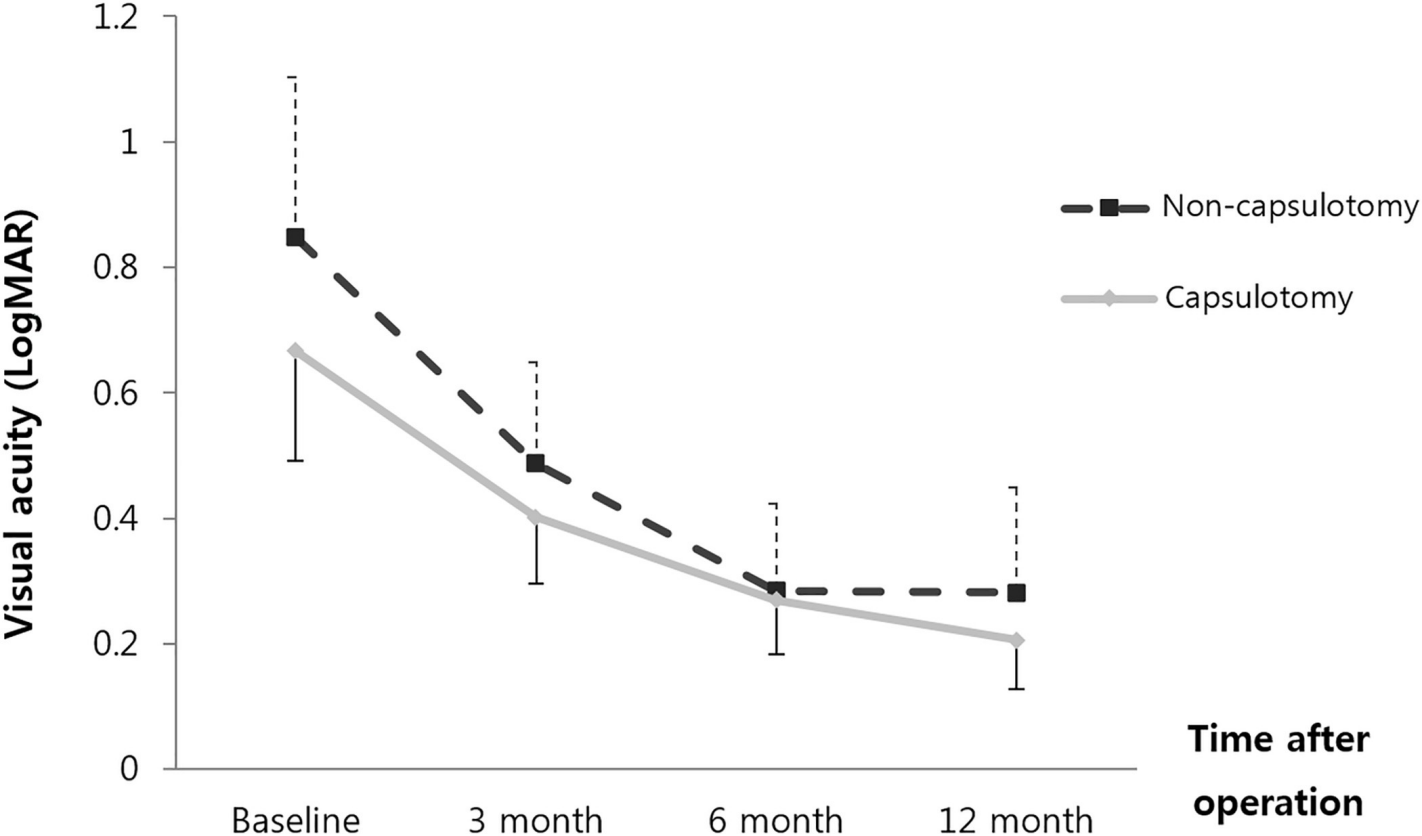
The change in best-corrected visual acuity(BCVA) in all 107 patients after combined phaco-vitrectomy for rhegmatogenous retinal detachment. Unmodified BCVA was no statistically significant difference between the two groups for all periods. The error bars represent the mean ± standard deviation.

**Fig 4:**
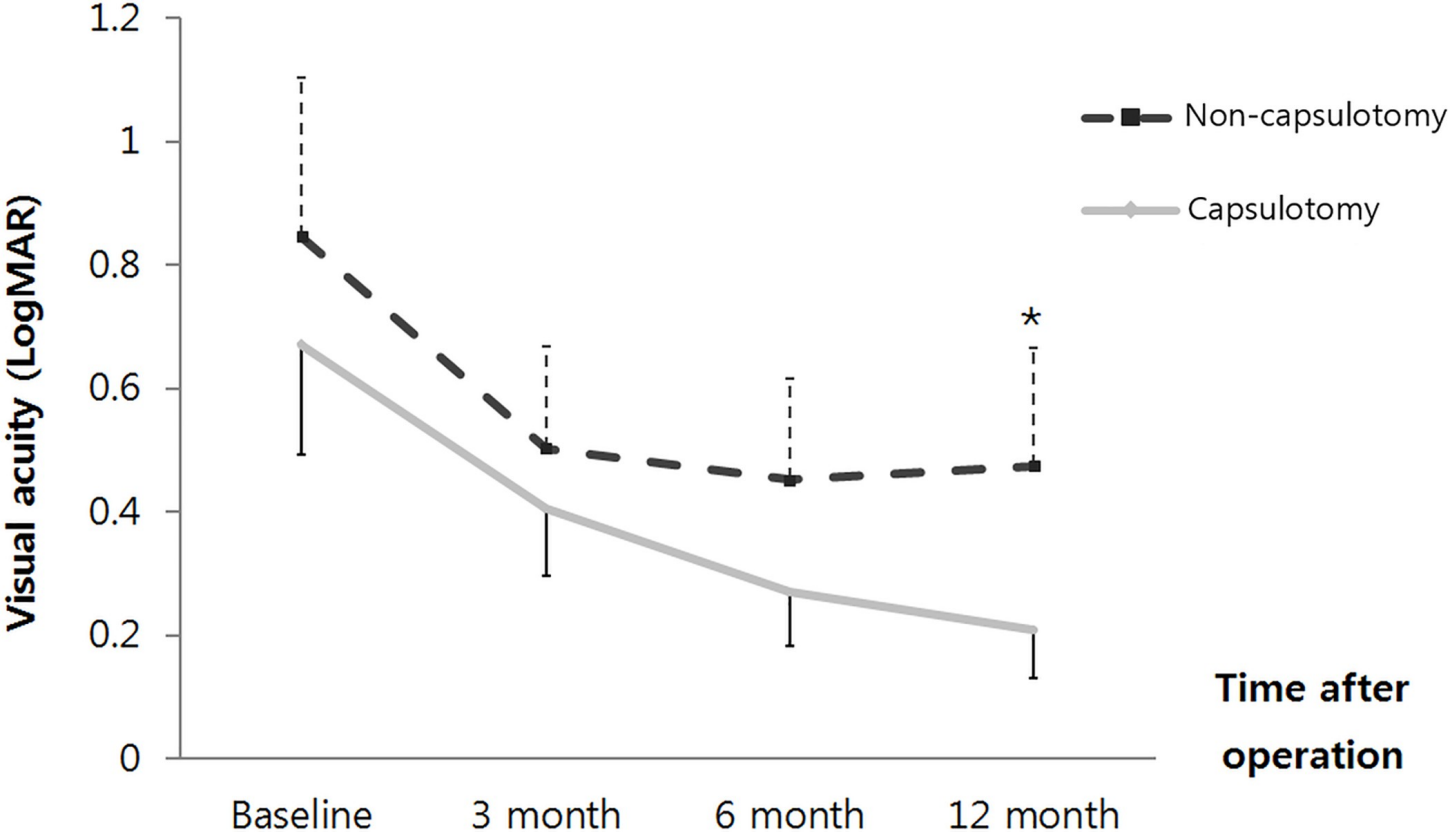
The change in modified best-corrected visual acuity(BCVA) in all 107 patients after combined phaco-vitrectomy for rhegmatogenous retinal detachment. In the group treated with capsulotomy, the mean modified BCVA was significantly better than that in the group not treated with capsulotomy at 12 months (*p = 0.014, independent *t*-test). The error bars represent the mean ± standard deviation.

**Fig 5:**
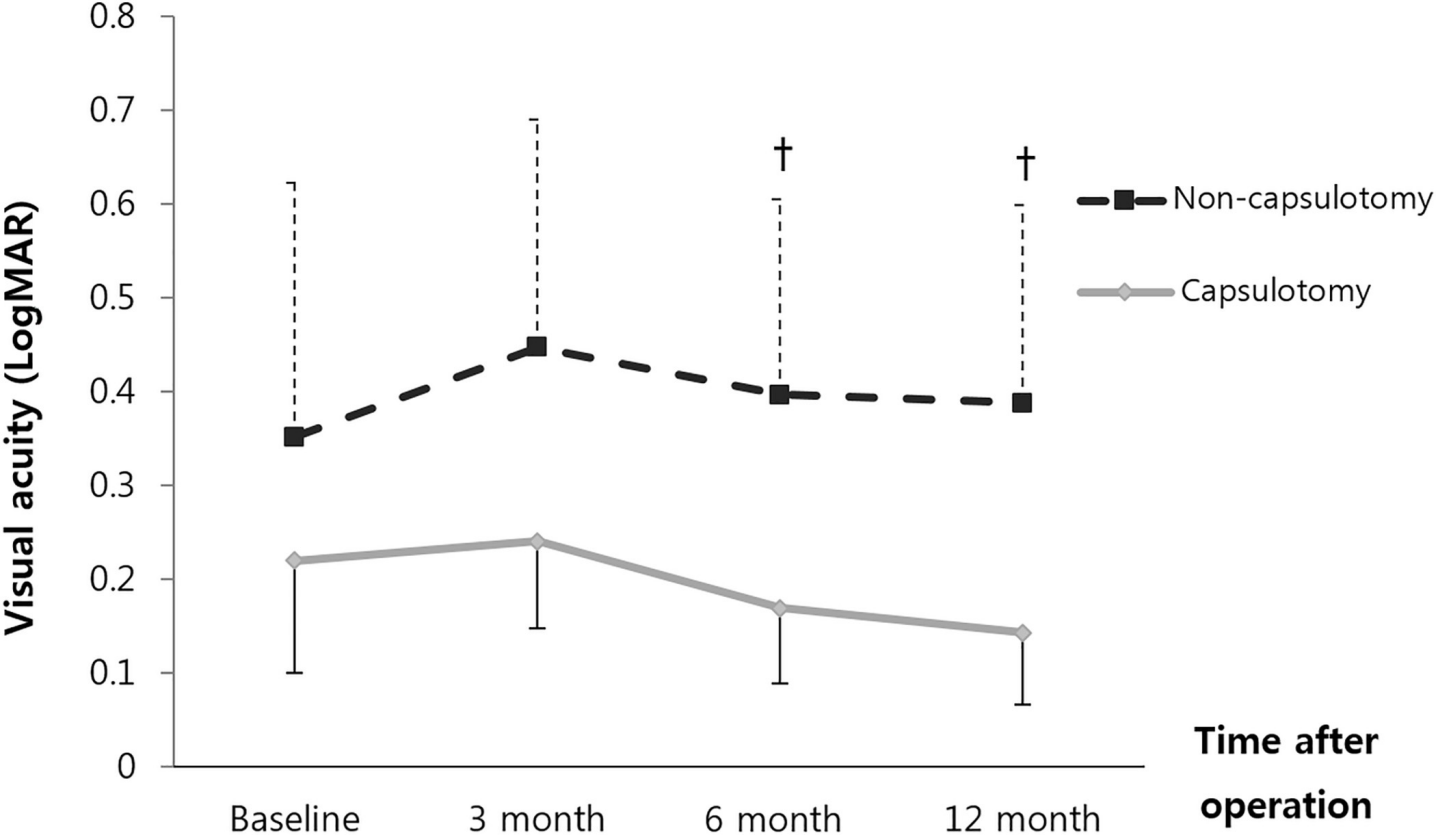
The modified best-corrected visual acuity(BCVA) change in 53 macular-on patients at the first visit after combined phaco-vitrectomy for rhegmatogenous retinal detachment. In the group treated with capsulotomy, the mean modified BCVA was significantly better than that in the group not treated with capsulotomy at postoperative 6 months (^†^p = 0.037; Mann-Whitney U test) and postoperative 12 months (^†^p = 0.030; Mann-Whitney U test). The error bars represent the mean ± standard deviation.

The BCVA was converted into logMAR values to compare the two groups. Both groups showed better visual acuities with time. When analyzing all of the patients, the BCVA of Group A and Group B were 0.67 ± 0.74 versus 0.85 ± 0.81 at the first visit(p = 0.258), 0.40 ± 0.45 versus 0.49 ± 0.51 at postoperative 3 months(p = 0.394), 0.27 ± 0.36 versus 0.28 ± 0.44 at postoperative 6 months(0.855), and 0.21 ± 0.33 versus 0.28 ± 0.53 at postoperative 12 months(p = 0.444), respectively(Fig 3). In unmodified BCVA, there was no statistical significance for all follow-up periods. However Modified BCVA of Group A and Group B were 0.40 ± 0.45 versus 0.50 ± 0.52 at postoperative 3 months(p = 0.309), 0.27 ± 0.36 versus 0.45 ± 0.51 at postoperative 6 months(0.055), and 0.21 ± 0.33 versus 0.47 ± 0.60 at postoperative 12 months(p = 0.014), respectively(Fig 4). The visual acuity of Group A was significantly better than that in Group B at postoperative 12 months. We analyzed 53 patients whose macula were preserved, involving 35 eyes in Group A and 18 eyes in Group B. The modified BCVA of Group A and Group B was 0.22 ± 0.36 versus 0.35 ± 0.58 at the first visit(p = 0.68), 0.24 ± 0.28 versus 0.45 ± 0.52 at postoperative 3 months(p = 0.294), 0.17 ± 0.24 versus 0.40 ± 0.45 at postoperative 6 months(p = 0.037), and 0.14 ± 0.23 versus 0.39 ± 0.45 at postoperative 12 months(p = 0.030), respectively. At 6 months and 12 months, the visual acuity of group A was significantly better than that in Group B (Fig 5).

### Complications

The patients did not experience any significant complication during surgery, and malpositioning including IOL dislocation was not detected. There were postoperative complications such as temporarily increased intraocular pressure, vitreous hemorrhage, recurrence of RD, posterior synechia, and epiretinal membrane, but there were no significant differences between the two groups (p > 0.05; Table 3).

**Table 3:** A comparison of postoperative complications in the two groups.

Number (%)	Group A (n = 68)	Group B (n = 39)	*P*-value
Transient IIOP	17(25.0%)	12(30.8%)	0.518*
Pupillary capture	0(0.0%)	2(5.1%)	0.131^†^
Vitreous hemorrhage	1(1.5%)	1(2.6%)	1.000^†^
Reccurence of RD	2(2.9%)	1(2.6%)	1.000^†^
Posterior synechiae	8(11.8%)	8(20.5%)	0.222*
ERM	8(11.8%)	5(12.8%)	0.872*
IOL decentration	0(0.0%)	0(0.0%)	1.000^†^

* Chi-square test

^†^ Fisher’s exact test

IIOP = increased intraocular pressure

RD = retinal detachment

ERM = epiretinal membrane

IOL = intraocular lens

## Discussion

This study analyzed the efficacy and usefulness of primary posterior capsulotomy using a 23-gauge vitreous cutter during combined phaco-vitrectomy in rhegmatogenous RD patients with a high risk of PCO. The mean modified BCVA in macular-on group was significantly better in the capsulotomy group than in the non-capsulotomy group at 6 and12 months. The poorer vision in the non-capsulotomy group was attributable to higher incidence of PCO. The incidence of PCO was 6.4-fold higher in the non-capsulotomy group than in the capsulotomy group (28.2% versus 4.4%). The incidence of PCO in our study was approximately 2-fold higher than that (12.5%) reported by Roh *et al*.[22] who conducted a study of patients with various retinal diseases who underwent combined phaco-vitrectomy. The differences was that our study only reported rhegmatogenous RD patients who were placed in a face-down position after gas tamponade and who were followed up for 1 year. Scharwey *et al*.[23] reported that combined surgery with intraocular air/gas tamponade induced severe posterior capsular fibrosis in the early postoperative period, and that was presumably caused by the accumulation of fibrin and proliferation stimulating factors in the narrow space between the IOL and the air/gas bubble. We hypothesize that a prolonged postoperative face-down position and an exudative membrane formed by inflammation associated with the intraocular laser might play a synergistic role in the development of PCO.

The etiology of PCO is unknown, but it is generally accepted that a number of factors such as capsulorhexis size and IOL material and design are involved in its pathogenesis.[24–26] PCO usually does not occur after removal of the posterior capsular bag and vitreous body, because there is no scaffold where lens epithelial cells can grow and proliferate on the capsule. However as the lens epithelial cells migrate to the IOL posterior surface, there is a possibility that PCO may occur even after capsulotomy. We called this earlier as a thin fibrotic membrane. Thin fibrotic membrane occured in only 3 of 68 patients in group A, which required extra capsulotomy using YAG laser, and all cases were younger patients under the age of 40. Because the capacities of cell division/regeneration and migration in young patients are better than those in elderly patients, the proliferation and migration of the lens epithelial cells might easily occur.[27]

In our study, most of the PCO was not observed in primary capsulotomy group during the one-year follow-up period. The reason for this is probably because the patient have no vitreous scaffold after total vitrectomy and had no past history of inflammation.

PCO is the most common complication of cataract surgery.[1, 2] The primary treatment option is Nd:YAG laser capsulotomy. The reported frequency ranges from 8.5% within 3 years after cataract extraction to 14.5% within 5 years. The incidence of PCO of combined phaco-vitrectomy is higher than that of cataract surgery only, because the former induces more inflammation.[15, 17, 22, 28] As described earlier, there are many possible complications in the YAG laser. The primary posterior capsulotomy at the time of the combined phaco-vitrectomy can prevent later PCO, be free from the laser-related complications, and save time and cost of follow-up after Nd:YAG laser capsulotomy. Complications of posterior capsulotomy were observed and compared in the two groups (Table 3), but no clinically significant complications were observed.

Gimbel[29] suggested that posterior CCC could be used in adult patients, although this surgical technique has been widely used in pediatric cataract surgery. A single posterior CCC or posterior optic buttonholing with optic capture of the IOL was also reported to prevent PCO.[11] Menapace[13] reported a lower incidence of complications in 1,000 consecutive patients after posterior optic buttonholing through a posterior CCC. However, the main disadvantages of this technique included the fact that an inexperienced surgeon may face a steep learning curve; if symmetrical patterns are not created in the center of capsular bag, poor centration can occur; the procedure is time consuming; and the results are not always reproducible.

The advent of small gauge vitrectomy has facilitated sutureless vitrectomy using smaller and thinner instruments, compared to the 20-gauge technique. In addition to reduced inflammation and more rapid visual recovery after surgery, a lower incidence of PCO has been reported with the smaller 23-gauge than the 20-gauge surgery.[30] Recent advances in vitrectomy cutter technology have also occurred, leading to development of the 7,500 cpm cutter.[31] In combined phaco-vitrectomy using the 25-gauge vitrectomy probe that is easily manipulated with a higher high cutting rate, posterior capsulotomy has been performed with better outcomes. Sato *et al*.[20] reported that the 25-gauge probe was advantageous for inexperienced surgeons who could perform a well-centered posterior capsulotomy in a desired size with less incidence of complications for a follow-up period of 1 year, as in our study, with the prevention of postoperative PCO. Aizawa *et al*.[21] also reported the advantages of 25-gauge posterior capsulotomy, although some differences from our study regarding a short follow-up period of 6 months that involved various retinal diseases and the use of several different IOL.

We found that primary posterior capsulotomy could be performed in an easier and simpler manner. A vitreous cutter was placed at the center of the capsular bag for proper positioning, and the stability of the IOL and the posterior capsulotomy were facilitated using a circular procedure, thereby completing the procedure with a slightly smaller incision than with an anterior CCC.

Schweitzer *et al*.[32] reported a myopic shift after combined phaco-vitrectomy with gas tamponade, because the gas bubble pushed the IOL forward during and after surgery, and the slight anterior displacement of the IOL resulted in a myopic shift. In contrast, there was no repositioning due to IOL decentration and no gas or air bubble was found in the anterior chamber after surgery in our study. For pupillary optic capture of the IOL, complications associated with the anterior displacement of the IOL were only observed in the non-capsulotomy group (two patients). For better IOL stability, the CCC size was important. In our study, the surgeon performed a 4.5 mm diameter CCC at the center of the anterior capsule, which minimized the possibility of inducing any severe complications. This diameter was slightly smaller than the size performed in routine cataract surgery.

Several factors have been reported that influenced the development of PCO, including the contact of gas or air with the posterior capsule, the rhegmatogenous RD itself, the presence or absence of diabetes[17], and the patient’s postoperative position. Rahman *et al*.[18] reported that the incidence of PCO was approximately 20.8% (46 of 221 eyes) using 23-gauge transconjunctival phaco-vitrectomy, and the main risk factors for PCO included rhegmatogenous RD (relative risk: 3.3), an axial length > 24.5 mm (relative risk: 2), gas tamponade (relative risk: 2.8), and a postoperative face-down position (relative risk: 4.1). Kim *et al*.[19] reported that the risk factors associated with PCO development after combined phaco-vitrectomy included younger age, CCC size, the combined treatment technique itself, and the injection of gas or silicone oil. Because rhegmatogenous RD patients simultaneously have several risk factors for PCO development, the incidence of PCO in this population might be higher than in patients with other retinal disease. Therefore, primary posterior capsulotomy should be very useful for patients with rhegmatogenous RD. It was difficult to accurately determine the incidence of PCO with regard to posterior capsulotomy, because various retinal diseases were included in previous studies with several types of IOL materials. Our study only reviewed the medical charts of rhegmatogenous RD patients who were operated by a single experienced surgeon, using the same instruments and one type of IOL, to monitor the incidence of PCO according to posterior capsulotomy and the visual loss associated with PCO development.

This study had a couple of limitations. First, we could not use actually measured BCVA and we used modified BCVA, because patients showing clinically advanced PCO were rapidly treated using Nd:YAG laser capsulotomy. Therefore, we had to follow the BCVA for 1 year based on the visual outcome data just before Nd:YAG laser. This was not a real visual acuity, but it was a concept which was inevitably introduced because of the need to compare visual acuity between the two groups to show the usefulness of the technique. Second, this retrospective study involved a non-prospective design that did not enable us to randomize the patients into capsulotomy or non-capsulotomy groups and because of the small number and retrospective study design, there is a possibility of selection bias.

In conclusion, our study showed that primary posterior capsulotomy using a 23-gauge vitrectomy cutter is a safe and useful surgical procedure that can be easily performed and does not have serious complications in rhegmatogenous RD patients. Thin fibrotic membrane was occured in some patients who underwent posterior capsulotomy and YAG laser treatment was needed. However this technique was able to prevent PCO, which was caused by high probability in rhegmatogenous RD patients undergoing the combined phaco-vitrectomy.

## Supporting information

S1 Dataset Clinical data, XLS format. This XLS include the patient's characteristics, visual acuities, complications.
(XLS)
